# Cannabidiol selectively attenuates lipotoxic immunometabolic inflammation in human macrophages

**DOI:** 10.3389/fimmu.2026.1873494

**Published:** 2026-07-16

**Authors:** Mayte Rueda-Munguía, Roberto Rodriguez-Moncayo, Luis Alberto Luévano-Martínez, Gerardo García-Rivas, Elena Cristina Castillo, Omar Lozano

**Affiliations:** 1 Tecnologico de Monterrey, Escuela de Medicina y Ciencias de la Salud, Monterrey, NL, Mexico; 2 Tecnologico de Monterrey, Institute for Obesity Research, Monterrey, Monterrey, NL, Mexico; 3 Hospital Zambrano Hellion, Unidad cardiometabólica Miguel y Betty Schwarz, Monterrey, NL, Mexico

**Keywords:** cannabidiol, immunometabolic, macrophages, palmitic acid, triglycerides

## Abstract

The role of saturated fatty acid-induced immunometabolic stress in macrophage dysfunction during metabolic disease remains incompletely understood, particularly the interplay between inflammatory signaling and intracellular lipid handling. We employed a tightly controlled palmitic acid (PA)-based lipotoxicity model in PMA-differentiated U937-derived human macrophage-like cells to investigate how lipid excess reshapes inflammatory responses and to evaluate the modulatory effects of cannabidiol (CBD). PA exposure induced a metabolically stressed yet viable macrophage phenotype, characterized by a broad cytokine remodeling profile. This included induction of classical proinflammatory cytokines such as interleukin (IL)-6, together with activation of inflammasome-associated cytokines IL-1β and IL-18 and additional immunoregulatory mediators, while tumor necrosis factor alpha (TNF-α) contributed to the overall inflammatory profile in a multivariate analysis. These changes were accompanied by a significant, time-dependent storage of intracellular triglycerides (TG) consistent with lipid overload and altered lipid handling. CBD co-treatment did not compromise cell viability but selectively attenuated PA-induced inflammatory response in a cytokine-dependent manner, with the most significant reduction observed at higher concentrations. In parallel, CBD significantly reduced intracellular TG accumulation under lipotoxic conditions. Collectively, these findings define a lipotoxicity-associated macrophage phenotype driven by saturated fatty acids and identify CBD as a context-dependent modulator of immunometabolic inflammation. This work provides a controlled experimental framework to study lipid-driven inflammatory dysfunction and supports the potential of CBD as a targeted strategy to modulate metabolic inflammation without broadly suppressing immune function.

## 1 Introduction

The immune system is increasingly recognized as a proactive regulator of metabolic homeostasis rather than merely a passive responder to infection. In this context, macrophages are important for bringing together immunometabolic signals. These cells detect changes in the local metabolic environment, such as increased fatty acids, altered glucose flux, or hypoxic stress, and convert these signals into specific transcriptional and signaling pathways, including NF-κB activation, endoplasmic reticulum stress signaling, and metabolic reprogramming. This dynamic sensing enables macrophages to support tissue adaptability under physiological conditions and positions them as principal contributors to chronic low-grade inflammation in the presence of sustained nutritional excess (1–3). Under physiological conditions, coupling of macrophages to nutrient levels facilitates tissue adaptation and repair; however, in the context of nutrient excess, this coupling becomes maladaptive, resulting in persistent elevation of pro-inflammatory mediators (4). This pattern characterizes low-grade metabolic dysfunction and contributes to metabolic and cardiometabolic disorders by promoting insulin resistance, tissue remodeling, and vascular inflammation (5, 6).

Among metabolic stressors, saturated fatty acids have been identified as significant contributors to immunometabolic dysregulation (7). Prolonged exposure of macrophages to high concentrations of palmitic acid (PA), a principal saturated fatty acid associated with obesity, elicits an inflammatory response characterized by a selective cytokine secretion profile dominated by canonical pro-inflammatory mediators such as tumor necrosis factor alpha (TNF-α), interleukin-1 beta (IL-1β), interleukin-6 (IL-6), and monocyte chemoattractant protein-1 (MCP-1) (3, 7). Unlike acute microbial signals, such as lipopolysaccharide (LPS), which induce rapid, typically transient, cytokine release, lipid overload imposes persistent metabolic stress that gradually alters macrophage function (1). This distinction is especially pertinent in metabolic disorders, where immune cells experience chronic rather than transient stimuli that, unlike those during infection, impair inflammation resolution.

A characteristic feature of PA-induced macrophage activation is the close association between signaling pathways governing TNF-α and IL-1β production and intracellular lipid metabolism. In adipose tissue, atherosclerotic plaques, and other lipid-rich pathological environments, macrophages accumulate neutral lipids and intracellular TG, resulting in lipid-laden or “foam-like” phenotypes (6, 8, 9). TG storage is typically regarded as an adaptive process that buffers excess free fatty acids by esterification and sequesters them within lipid droplets, thereby limiting the accumulation of cytotoxic lipid intermediates (8). Nevertheless, accumulating evidence suggests that persistent TG storage is not metabolically inactive (10). Lipid-laden macrophages exhibit disrupted lipid flux, altered mitochondrial metabolism, and a diminished ability to respond to additional metabolic stressors, features closely associated with sustained secretion of TNF-α and IL-1β and prolonged elevation of IL-6 and MCP-1 (9, 10).

Cannabidiol (CBD), a non-psychoactive phytocannabinoid, has emerged as a potential regulator of inflammatory and metabolic processes. Unlike traditional anti-inflammatory drugs, CBD exerts context-dependent immunomodulatory effects across multiple experimental systems, encompassing both immune and metabolic cells (11). CBD does not function as a general immunosuppressant; rather, its actions appear to depend on the type of activating stimuli, cellular metabolic state, and inflammatory environment (11, 12). This characteristic has positioned CBD as a potential regulator of maladaptive inflammation rather than a nonspecific inhibitor of immune function.

Despite growing interest in CBD, its effects on macrophage immunometabolism under conditions of lipid excess remain incompletely characterized. Most prior research has examined CBD in models of acute LPS-induced inflammation or oxidative stress, with limit attention to the combined metabolic and inflammatory burden imposed by saturated fatty acids (13). Furthermore, the extent to which CBD affects intracellular lipid handling, particularly TG accumulation under lipotoxic conditions, remains poorly investigated in human macrophages (11, 14). These gaps are particularly relevant given that, in metabolic and cardiometabolic diseases, macrophage activation is primarily driven by nutrient excess rather than infection, with TNF-α, IL-1β, IL-18, IL-6, and MCP-1 acting as key mediators of tissue dysfunction (6, 15).

This work investigated whether lipid-driven stress induces a distinct immunometabolic state in PMA-differentiated U937-derived human macrophage-like cells and whether this response can be selectively modulated by CBD. We hypothesized that PA exposure drives a coordinated reprogramming characterized by specific pro-inflammatory cytokine release and intracellular TG accumulation and that CBD attenuates these maladaptive responses in a context-dependent manner without compromising cell viability.

## 2 Materials and methods

### 2.1 Reagents

PA (PA; Sigma-Aldrich, Cat. 1001581306; CAS 57-10-3) was conjugated to fatty acid-free bovine serum albumin (BSA) at a defined molar ratio to generate a PA-BSA complex. The mixture was incubated at 37 °C for 1 hour and adjusted to pH 7.4. The resulting PA-BSA stock solution, containing 1 mM PA and 0.17 mM BSA, was used to achieve a final working concentration of 200 µM PA and 0.033 mM BSA, corresponding to a 6:1 molar ratio. PA-BSA stocks were sterile-filtered, aliquoted, and stored at -20 °C. Prior to use, aliquots were thawed once and diluted immediately in a complete RPMI 1640 culture medium. Control conditions received an equivalent concentration of BSA without PA.

High purity cannabidiol (CBD; ≥98% purity) was solubilized in DMSO and used at final concentrations of 0.01, 0.1, 1, 5, and 10 µM. The final DMSO concentration was maintained at 0.1% (v/v; 1 µL/mL) across all experimental conditions, including controls. Lipopolysaccharide (LPS; Merck, L5886) was resuspended in sterile PBS to a final concentration of 50 ng/mL.

### 2.2 Cell culture and macrophage differentiation

U937 human monocytes (ATCC, CRL-1593.2) were cultured in RPMI 1640 medium supplemented with 10% heat-inactivated fetal bovine serum (FBS), 1% penicillin-streptomycin, and sodium bicarbonate (2 g/L) at 37 °C in a humidified atmosphere containing 5% CO_2_. Macrophage-like differentiation was induced by treatment with phorbol 12-myristate 13-acetate (PMA; 50 nM). Following differentiation, cells were rinsed twice with sterile PBS to remove residual PMA and non-adherent cells. Experimental interventions were initiated immediately after PBS washing.

### 2.3 Experimental design and treatment conditions

For metabolic activity and viability, differentiated macrophages were seeded at a density of 40,000 cells/well in 200 µL of complete medium in 96-well plates. For cytokine profiling and TG quantification, cells were cultured in 12-well plates at a density of 0.5 × 10^6^ cells/well in 1 mL of complete medium. Cells were adherent at the time of treatment initiation. Peripheral wells were excluded to avoid edge effects.

The experimental groups comprised (i) CTRL, consisting of BSA- and DMSO-matched vehicle controls; (ii) PA, treated with palmitic acid (200 µM for 24 hours, unless otherwise indicated in the figure legends); (iii) PA + CBD, co-treated with PA (200 µM) and CBD (0.01–10 µM) for 24 hours; (iv) LPS, treated with LPS (50 ng/mL) for 24 hours; and (v) LPS + CBD, co-treated with LPS (50 ng/mL) and CBD (1 or 10 μM) for 24 hours. Co-treatments were administered concurrently at the initiation of the treatment period.

### 2.4 Metabolic activity assay

Alamar Blue reagent (10% v/v) was added directly to the culture medium and incubated for 3 hours at 37 °C. Fluorescence intensity was measured at 540–570 nm excitation wavelengths and 580–610 nm emission wavelengths. Data were expressed as a percentage of fluorescence normalized to the control, which was set to 100%.

### 2.5 Cell viability assay

Cells were rinsed with PBS, fixed with 4% paraformaldehyde for 15 minutes, and stained with 0.5% crystal violet for 30 minutes at room temperature. After staining, wells were rinsed with distilled water to remove excess dye and then air-dried. The bound dye was solubilized in 100% isopropanol, and absorbance was measured at 592 nm. Cell viability was expressed as a percentage relative to the control, which was set to 100%.

### 2.6 Intracellular TG quantification

Cells were lysed in a buffer containing 50 mM Tris-HCl (pH 7.4) and 1% NP-40 detergent for 30 minutes at room temperature, scraped, and transferred to microtubes. Lysates were heated at 50 °C for 10 minutes to facilitate lipid solubilization and centrifuged to remove insoluble material. TG content in the supernatants was quantified using an enzymatic colorimetric assay (SpinReact), with absorbance measured at 505 nm. TG levels were normalized to total protein content, determined by the Lowry assay, and expressed as µg TG/µg protein. For kinetic experiments, TG were measured at 2, 6, 12, and 24 hours after treatment initiation.

### 2.7 Cytokine profiling

Cell culture supernatants were harvested after 24 hours of treatment, centrifuged at 500 x g for 5 minutes to remove cell debris, and stored at -80 °C until analysis. Cytokine concentrations were quantified using the LEGENDplex™ Human Inflammation Panel (13-plex; BioLegend), which measures IL-1β, IL-6, IL-8, IL-10, IL-12p70, IL-17A, IL-18, IL-23, IFN-α, IFN-γ, TNF-α, MCP-1, and IL-1RA. Samples were processed according to the manufacturer’s guidelines and acquired on a CytoFLEX Beckman cytometer (40,000 events per sample). FCS files were analyzed using FlowJo (BD Biosciences) with gating according to the manufacturer’s instructions. Median fluorescence intensity (MFI) values were used to generate standard curves using a four-parameter logistic regression model in Prism (GraphPad). Cytokine concentrations from experimental samples were determined by interpolation from the corresponding standard curves.

### 2.8 Statistical analysis

Data are expressed as mean ± SEM. Group comparisons were performed using either one-way analysis of variance (ANOVA) followed by Dunnett’s *post hoc* test or two-way ANOVA (factors: treatment and time) followed by Tukey’s *post hoc* test. Statistical significance was established at p < 0.05.

### 2.9 Principal component analysis

Cytokine concentration values were compiled across all experimental conditions and imported into a custom MATLAB script (MathWorks) for analysis. Principal component analysis (PCA) was performed on the cytokine concentration matrix to identify dominant sources of variance across experimental conditions. Separate PCA analyses were performed for each stimulus (LPS and PA) to assess cytokine response and its modulation by CBD. PCA scores were used to visualize sample separation in reduced-dimensional space, and loading vectors were used to evaluate the contribution of individual cytokines to each principal component. For each condition, PCA scores were calculated as the mean of n=2 technical replicates across 5 independent experiments. PCA plots were generated in MATLAB.

## 3 Results

### 3.1 Palmitic acid induces metabolic impairment and lipid accumulation, partially counteracted by cannabidiol, in U937-derived macrophages

**Figure 1:**
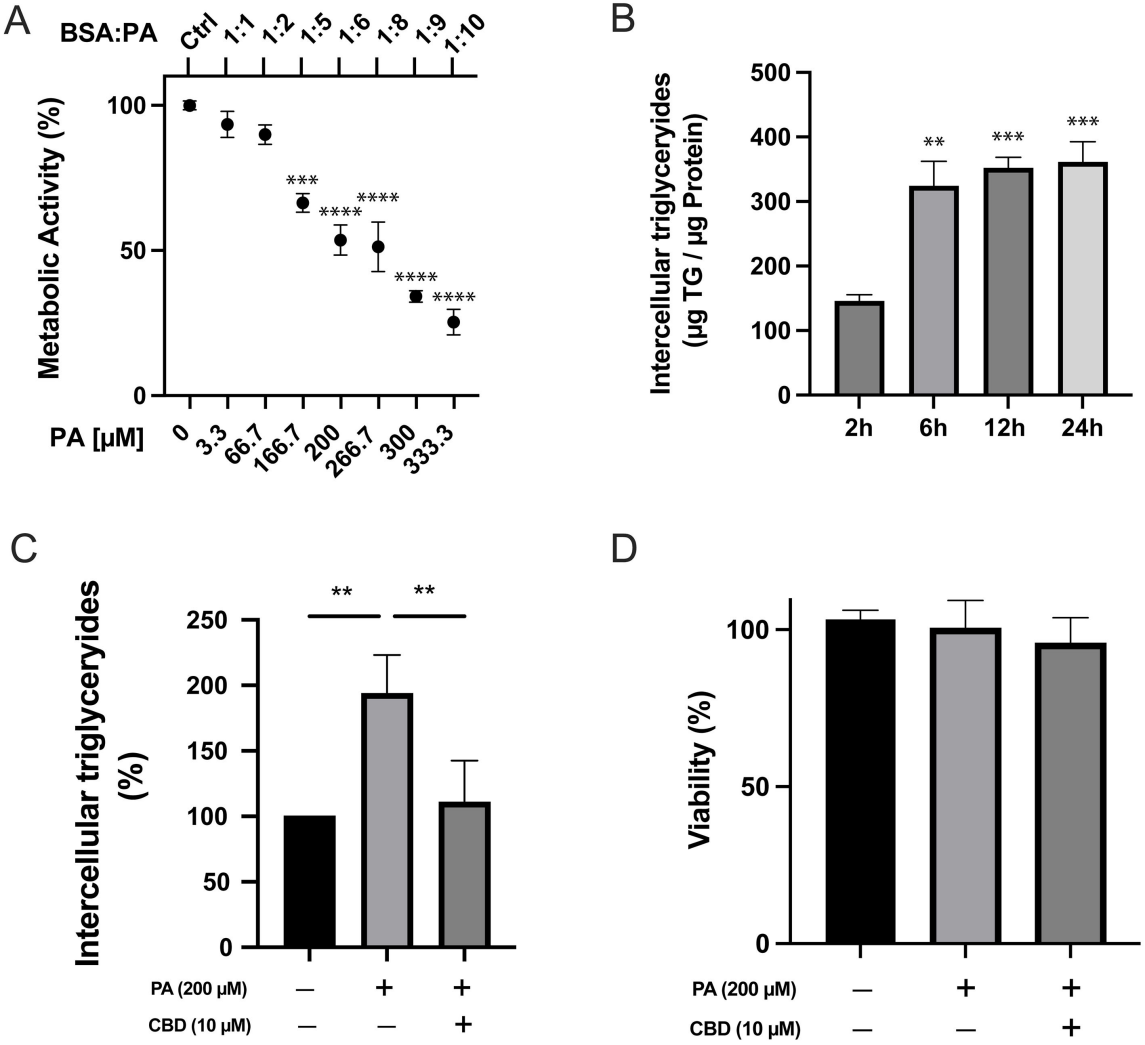
PA induces metabolic dysfunction and lipid accumulation in U937-derived macrophages, partially reversed by cannabidiol. Human macrophages differentiated from U937 cells were exposed to increasing PA. **(A)** Metabolic activity was evaluated after exposure to increasing PA concentrations complexed with BSA at different BSA: PA molar ratios. (n=3) **(B)** Intracellular triglyceride content was measured over time following PA exposure from 2h to 24 h. (n=4) **(C)** Triglyceride accumulation under selected conditions (PA 200 μM ± CBD 10 μM). (n=3) **(D)** Cell viability by crystal violet under selected conditions (PA 200 μM ± CBD 10 μM) (n=5). Data are presented as the mean ± SEM, calculated from three independent experiments. Statistical significance was assessed using one-way analysis of variance (ANOVA) with Tukey’s *post hoc* test for panel **(C)**, and Dunnet’s *post hoc* test for panels **(A, B, D)**. **P ≤ 0.01; ***P ≤ 0.001, and ****P ≤ 0.0001.

Exposure to elevated PA: BSA ratios led to a gradual reduction in metabolic activity in U937-derived macrophages, consistent with a dose-dependent reduction in metabolic activity associated with lipotoxic stress (**Figure 1A**). Notably, the 1:6 PA: BSA ratio (200 μM) produced a reduction in metabolic activity close to 50% relative to control conditions (p < 0.0001) and was therefore selected for subsequent experiments as it approximated the half-maximal inhibitory concentration (IC50). Under this selected condition, exposure to PA (200 μM) promoted a marked accumulation of intracellular triglycerides over time, with significant increases observed from 6 h (p = 0.0012) and sustained up to 24 h (**Figure 1B**), indicating altered lipid handling under lipid excess. In this context, co-treatment with CBD (10 μM) markedly decreased (p = 0.0042) intracellular triglyceride accumulation relative to PA-treated cells (**Figure 1C**), indicating a modulatory influence of CBD on lipid metabolism. Importantly, triglyceride levels in the PA+CBD group did not differ significantly from those in the control conditions (p = 0.9702), indicating near-complete prevention of PA-induced lipid accumulation rather than a partial reversal. No significant differences in cell viability were observed among experimental conditions (**Figure 1D**).

### 3.2 CBD selectively attenuates the PA-induced inflammatory cytokine response in U937-derived human macrophages

**Figure 2:**
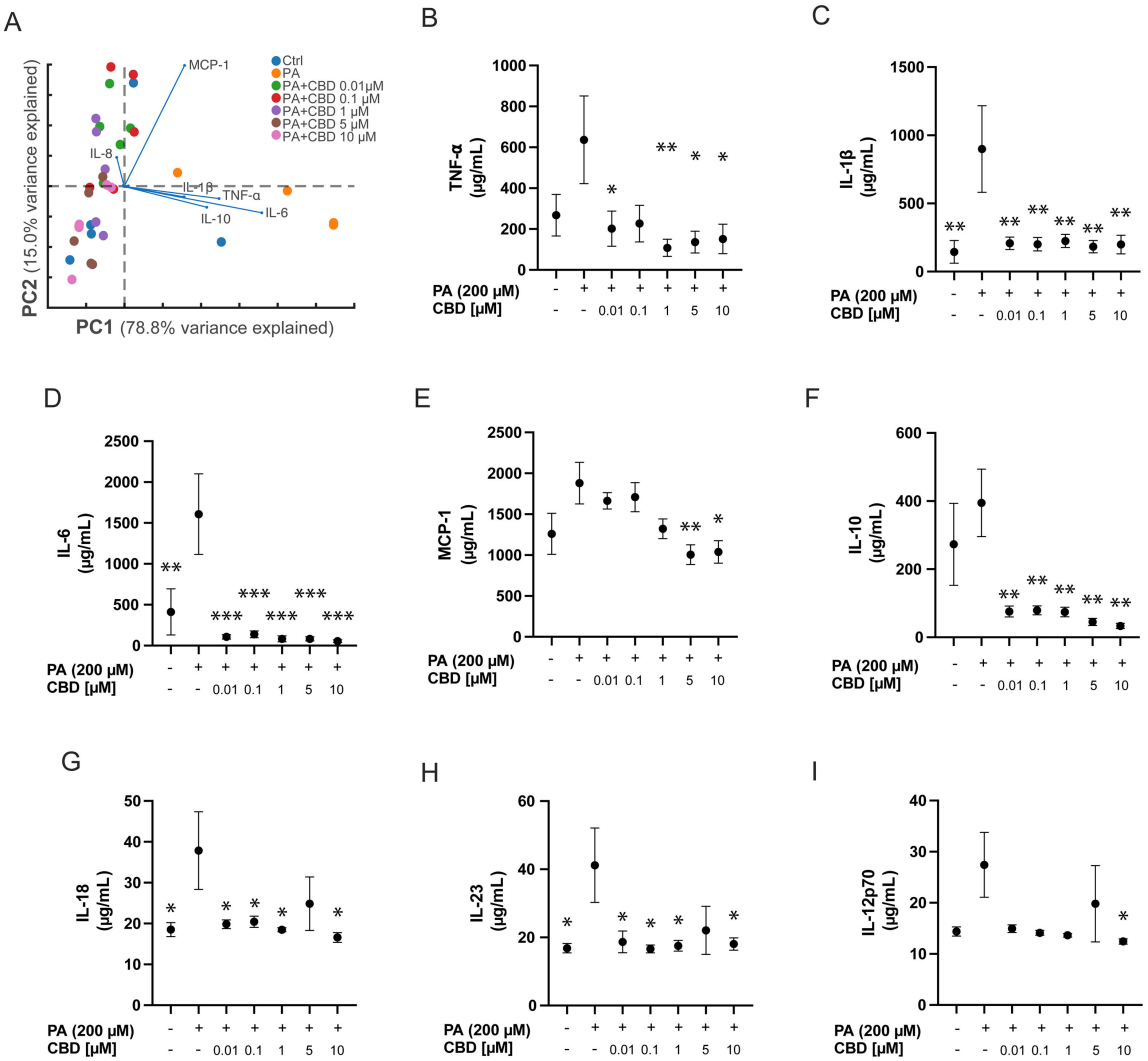
CBD impaired the PA-driven pro-inflammatory cytokine response. U937-derived human macrophages were exposed to 200 μM PA for 24 hours and co-treated with escalating doses of CBD. Cytokine secretion was measured in culture supernatants collected at 24 hours. **(A)** Principal component analysis (PCA) of cytokine secretion profiles. Score plots show the distribution of samples under control, PA, and PA+CBD conditions, while loading vectors indicate the contribution of individual cytokines to each principal component. PA induces broad dispersion across PC1 (78.8% variance explained) and PC2 (15.0% variance explained), which is progressively reduced by CBD. **(B–I)** Individual cytokine measurements corresponding to the dominant contributors identified by PCA: **(B)** TNF-α, **(C)** IL-1β, **(D)** IL-6, **(E)** IL-10, **(F)** IL-18, **(G)** IL-23, **(H)** IL-12p70, and **(I)** IL-33. Data are presented as mean ± SEM from five independent experiments with two biological replicates. Statistical significance was assessed by one-way ANOVA with Dunnett’s *post hoc* test. Comparisons are shown relative to the PA condition: *P ≤ 0.05; **P ≤ 0.01; ***P ≤ 0.001.

To define the structure of the inflammatory response induced by lipotoxic stress, we performed principal component analysis (PCA) on a panel of 13 cytokines (**Figure 2A**; **Supplementary Table 1**). PA-treated macrophages exhibited a wide distribution in the PCA plane relative to control conditions. CBD treatment progressively shifted samples toward the control samples with a concomitant reduction in their dispersion, suggesting a decrease in the inflammatory response across multiple cytokines. Through the loading vectors, we identified that PC1 is primarily driven by IL-6, TNF-α, and IL-1β, whereas PC2 was associated with MCP-1 and IL-18.

Based on PCA, we next examined individual cytokines that contributed most to the data variance, confirming that PA administration elicited an inflammatory response in U937-derived human macrophages. In the univariate analysis, TNF-α (**Figure 2B**) release upon PA exposure did not result in a statistically significant increase compared with control conditions (p=0.0991), although a trend toward higher levels was observed. Notably, CBD co-treatment significantly reduced TNF-α secretion relative to the PA group (p < 0.05), reaching levels comparable to basal conditions. Nonetheless, IL-1β levels exhibited a considerable elevation following PA exposure (p < 0.001) (**Figure 2C**), demonstrating an approximate 4-6-fold increase compared to control. The impact was significantly diminished by CBD in a dose-dependent manner, with reductions of approximately 70% relative to PA-treated cells at higher concentrations.

In addition, IL-6 secretion was significantly increased in response to PA (p < 0.0029). This increase was markedly attenuated by CBD across multiple concentrations (**Figure 2D**). CBD treatment reduced IL-6 levels consistently across concentrations, with reductions ranging between 45%–65% compared with PA-treated cells, indicating a sustained modulatory effect. A similar suppressive effect of CBD was observed for MCP-1, although the differences were more pronounced, with decreases around 80%–85% at the higher CBD concentrations (**Figure 2E**).

Conversely, IL-10 exhibited a distinct pattern of regulation. PA exposure significantly increased IL-10 secretion relative to control conditions (**Figure 2F**). CBD treatment reduced IL-10 levels across concentrations with decreases of approximately 60–80% relative to PA-treated cells, indicating a robust downregulation of this cytokine. Additional cytokines included in the expanded panel further supported a broad immunomodulatory effect of CBD. IL-18 secretion was significantly modified across treatment conditions, with CBD reducing PA-associated levels at selected concentrations, although this effect was not uniform across all doses (**Figure 2G**). Likewise, IL-23 was significantly decreased by CBD in several treatment groups, whereas the effect was lost at some intermediate or higher concentrations, suggesting a non-linear dose-response pattern (**Figure 2H**). In contrast, IL-12p70 was comparatively less responsive, with significant modulation evident only at the highest CBD concentration tested (**Figure 2I**).

### 3.3 Differential modulation of LPS-induced cytokine secretion by CBD in U-937-derived human macrophages

**Figure 3:**
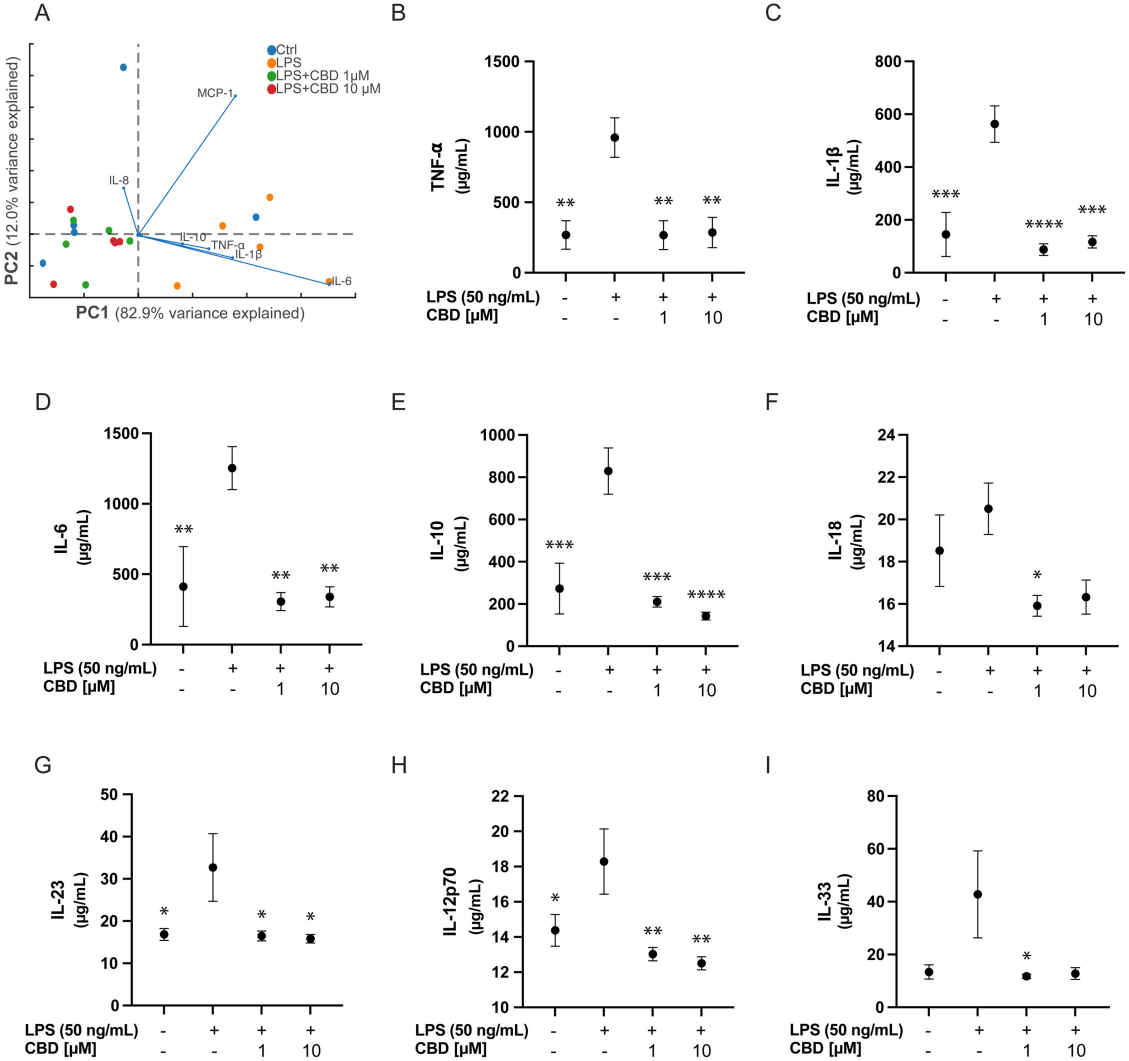
Effect of CBD on LPS-induced cytokine secretion by U937-derived human macrophages. U937-derived macrophages were stimulated with LPS (50 ng/mL) and co-treated with CBD (1 or 10 μM) for 24 hours. Cytokine levels were quantified in culture supernatants after 24 h. **(A)** Principal component analysis (PCA) of cytokine secretion profiles. PC1 (82.9% variance explained) and PC2 (12.0% variance explained). Score plots show the distribution of control, LPS, and LPS+CBD conditions, while loading vectors indicate the contribution of individual cytokines to each principal component. LPS induces a directional shift primarily along PC1, which is partially reversed by CBD. Individual cytokine measurements corresponding to the dominant contributors identified by PCA: **(B)** TNF-α, **(C)** IL-1β, **(D)** IL-6, **(E)** MCP-1, **(F)** IL-10, **(G)** IL-18, **(H)** IL-23, and **(I)** IL-12p70. Data are presented as mean ± SEM from five independent experiments with two biological replicates. Statistical significance was assessed by one-way ANOVA with Dunnett’s *post hoc* test. Comparisons are shown relative to the LPS condition: *P ≤ 0.05, **P ≤ 0.01, ***P ≤ 0.001, and ****P ≤ 0.0001.

Applying the same PCA framework as previously described to macrophages challenged with LPS (50 ng/mL), we observed a directional shift in the PCA plane of LPS-treated samples with respect to control conditions (**Figure 3A**). Loading vectors identified TNF-α, IL-1β, and IL-6 as the main contributors of this shift, suggesting a canonical TLR-4-mediated inflammatory response. Furthermore, CBD treatment shifted the samples toward the control space, indicative of an attenuated response.

Guided by PCA, we next examined individual cytokines, confirming that LPS stimulation induced a cytokine-specific pro-inflammatory response in U937-derived human macrophages. The response was characterized by significant increases in TNF-α, IL-1β, IL-6, IL-10, IL-23, and IL-12p70 relative to control cells (**Figures 3B–E, G–H**). In contrast, IL-18 and IL-33 did not show a significant increase in response to LPS alone under these experimental conditions (**Figures 3F, I**).

Co-treatment with CBD selectively modulated this response. TNF-α secretion was significantly reduced at both 1 μM and 10 μM CBD compared with the LPS group (**Figure 3B**). A similar suppressive effect was observed for IL-1β and IL-6, whose levels were markedly decreased at both CBD concentrations (**Figures 3C, D**). Likewise, CBD significantly reduced IL-10 secretion, indicating a modulatory effect beyond canonical pro-inflammatory cytokines and affected cytokines with regulatory or compensatory roles in the inflammatory response (**Figure 3E**).

Additional IL-23 and IL-12p70 were significantly decreased by CBD at both concentrations tested (**Figures 3G, H**). By contrast, IL-18 and IL-33 exhibited a more selective pattern, with significant reductions observed at 1 μM CBD but not at 10 μM, suggesting a non-linear dose-response for these mediators (**Figures 3F–I**).

## 4 Discussion

### 4.1 Palmitic acid-induced immunometabolic reprogramming in human macrophages

Chronic exposure to saturated fatty acids is increasingly recognized as a key driver of immunometabolic reprogramming in macrophages during metabolic diseases (1, 3, 5, 16). In this context, the use of palmitic acid (PA) at 200 μM under 24 h exposure conditions builds on prior observations from our group showing that this concentration induces lipotoxic stress without compromising macrophage viability (17). A 24 h exposure period was selected to characterize the immunometabolic response to PA, as macrophage cytokine secretion is known to occur rapidly following stimulation and often reaches robust levels within this timeframe (18). While longer exposure periods may provide additional insight into the persistence of these responses, the present study was designed to capture the immunometabolic effects of lipotoxic stress. This approach enables modeling of nutrient overload while preserving cellular functionality, thereby allowing downstream immunometabolic analyses. Importantly, PA was delivered as a fatty acid–free BSA-conjugated complex (1:6 molar ratio), a widely used approach to approximate physiological fatty acid transport and minimize non-specific toxicity.

In contrast to *in vivo* models of obesity, in which macrophages are exposed to multiple fatty acids, adipokines, and systemic metabolic cues (3, 5), this reductionist PA-based model enables a more direct attribution of cytokine remodeling to a defined lipotoxic stimulus, while acknowledging that it does not fully recapitulate the adipose tissue microenvironment.

At the inflammatory level, PA exposure induced a broad cytokine response, including increased secretion of IL-1β, IL-6, and MCP-1, together with differential modulation of IL-10, IL-18, IL-23, and IL-12P70. Although TNF-α did not reach statistical significance in univariate analysis, PCA identified it as a contributor to the overall inflammatory signature, suggesting a coordinated but modest involvement in PA-induced inflammatory response. This cytokine profile aligns with previous studies linking saturated fatty acids to activation of innate immune pathways in macrophages, including pathways previously associated with endoplasmic reticulum stress, NF-κB inflammatory signaling, and inflammasome-dependent cytokine maturation. The increased secretion of IL-1β and IL-18 supports the involvement of inflammasome-associated cytokine processing under lipotoxic conditions (7, 8, 19–21); however, the specific inflammasome complex and upstream molecular intermediates were not directly assessed in the present study.

Within this framework, metabolic alterations, reflected by decreased metabolic activity and TG accumulation, co-occurred with cytokine secretion, in agreement with lipid-induced innate immune activation described in settings of nutrient excess (1, 5, 22, 23). Together, these findings indicate that PA exposure promotes concurrent inflammatory and metabolic alterations characterized by cytokine remodeling and intracellular lipid accumulation in U937-derived macrophages.

### 4.2 Immunometabolic macrophage inflammation: differential cytokine programs and stimulus-dependent modulation by CBD

Building upon the cytokine profile described above, we next examined whether CBD differentially modulated inflammatory responses induced by lipotoxic and canonical inflammatory stimuli. Building upon the cytokine profile described above, we next examined whether CBD differentially modulated inflammatory responses induced by lipotoxic and canonical inflammatory stimuli. PA and LPS generated partially distinct cytokine profiles despite sharing induction of key inflammatory mediators. Whereas LPS stimulation produced a response dominated by TNF-α, IL-1β, and IL-6, PA elicited a broader immunometabolic inflammatory profile characterized by additional modulation of cytokines such as IL-18 and IL-23. These differences suggest that lipotoxic and endotoxin-driven activation engage overlapping but non-identical inflammatory programs (4, 24–26). Multivariate PCA further supported stimulus-specific cytokine signatures and revealed that CBD shifted cytokine responses toward the control space, consistent with a context-dependent modulatory effect.

Consistent with the broader cytokine activation observed under PA stimulation, CBD preferentially attenuated TNF-α and IL-1β, suggesting that its immunomodulatory effects are more evident in the context of lipid-driven immunometabolic activation. Taken together, these findings support a stimulus-dependent modulation of cytokine secretion by CBD rather than uniform suppression of inflammatory mediators. However, because upstream signaling intermediates were not directly assessed, these data should be interpreted as evidence of immunomodulation rather than direct demonstration of pathway-specific inhibition. Although the cytokine profile observed is consistent with inflammatory programs previously linked to NF-κB–associated signaling and inflammasome-dependent cytokine maturation, the present study did not evaluate specific upstream intermediates, such as p65 activation, IκBα degradation, ASC oligomerization, caspase-1 activation, or the identity of the inflammasome complex involved (11, 12, 27, 28).

This context dependency was further supported by differential cytokine modulation across stimuli. Under LPS stimulation, CBD significantly reduced IL-12p70 at 1 and 10 μM, whereas under PA conditions, this effect was only observed at the highest concentration tested (10 μM). Similarly, CBD reduced IL-18 more consistently under PA stimulation, while only a limited effect was observed under LPS. These findings suggest that lipid-driven and endotoxin-driven inflammatory states differ in their susceptibility to CBD-mediated modulation (29). Taken together, these findings support a stimulus-dependent effect of CBD on cytokine secretion, rather than a broad immunosuppressive action (11, 12, 27, 28).

Notably, previous studies reporting stronger anti-inflammatory effects of CBD under LPS stimulation have frequently been conducted in murine macrophage or microglial models (e.g., RAW264.7 and BV2) using higher concentrations (5-25 μM), which may enhance inhibitory effects but can also impact cell viability (30, 31). In contrast, the concentration range used in the present study was selected to preserve cellular functionality, which may contribute to the more selective cytokine modulation observed.

An additional observation was the non-linear modulation of selected cytokines, including IL-10, across the CBD concentration range. Such behavior is consistent with the well-described biphasic pharmacology of CBD, which has been reported to exhibit bell-shaped or non-monotonic dose-response relationships in several experimental systems (32). Furthermore, individual cytokines are regulated by partially distinct signaling and transcriptional pathways, which may contribute to the heterogeneous concentration-response patterns observed in the present study. Therefore, the observed responses may reflect the complex pharmacology of CBD rather than a strictly linear inhibitory effect.

The concentration range evaluated in this study (0.01–10 μM) was selected based on previous reports demonstrating immunomodulatory activity of CBD without significant cytotoxicity in immune cell models (33). Nevertheless, the direct translation of *in vitro* CBD concentrations to *in vivo* exposure levels remains challenging, since factors including bioavailability, metabolism, tissue distribution, and clearance influence effective drug concentrations. Our workgroup has previously shown that doses up to 10 mg/kg administered subcutaneously to a mice model of heart failure with reduce ejection fraction (HFrEF) can reduce inflammation and improve heart function (34). Nonetheless, there is still a need for future pharmacokinetic studies.

The present study was performed exclusively in PMA-differentiated U937-derived macrophages. Although this model is widely used and provides high experimental reproducibility for studying macrophage inflammatory and immunometabolic responses, validation in primary human monocyte-derived macrophages and *in vivo* models will be important to further assess the generalizability and translational relevance of the observed effects.

Overall, these findings position CBD not as a broad-spectrum anti-inflammatory agent, but as a context-dependent modulator of immunometabolic inflammation. This distinction is particularly relevant in metabolic diseases, where nutrient-driven inflammation coexists with the need to preserve host defense. By reducing selected cytokines and intracellular triglyceride accumulation under conditions of lipid excess, CBD emerges as a candidate molecule for further investigation in models of metabolic inflammation. However, additional mechanistic studies and validation in primary macrophages and *in vivo* systems will be necessary to determine its therapeutic relevance.

## 5 Conclusion

This research establishes a physiologically functional lipotoxic model in U937-derived human macrophages, in which PA elicits a significant pro-inflammatory and lipid-accumulating phenotype while maintaining cell survival. Under these conditions, PA significantly enhanced the release of IL-6, and MCP-1, with TNF-α contributing to a lesser extent. Furthermore, PA induced the secretion of cytokines associated with inflammasome activation and immunometabolism regulation, including IL-1β, IL-18, and IL-23, while promoting time-dependent intracellular triglyceride storage, consistent with concurrent inflammatory and lipid-storage alterations induced by saturated fatty acid exposure.

**Figure 4:**
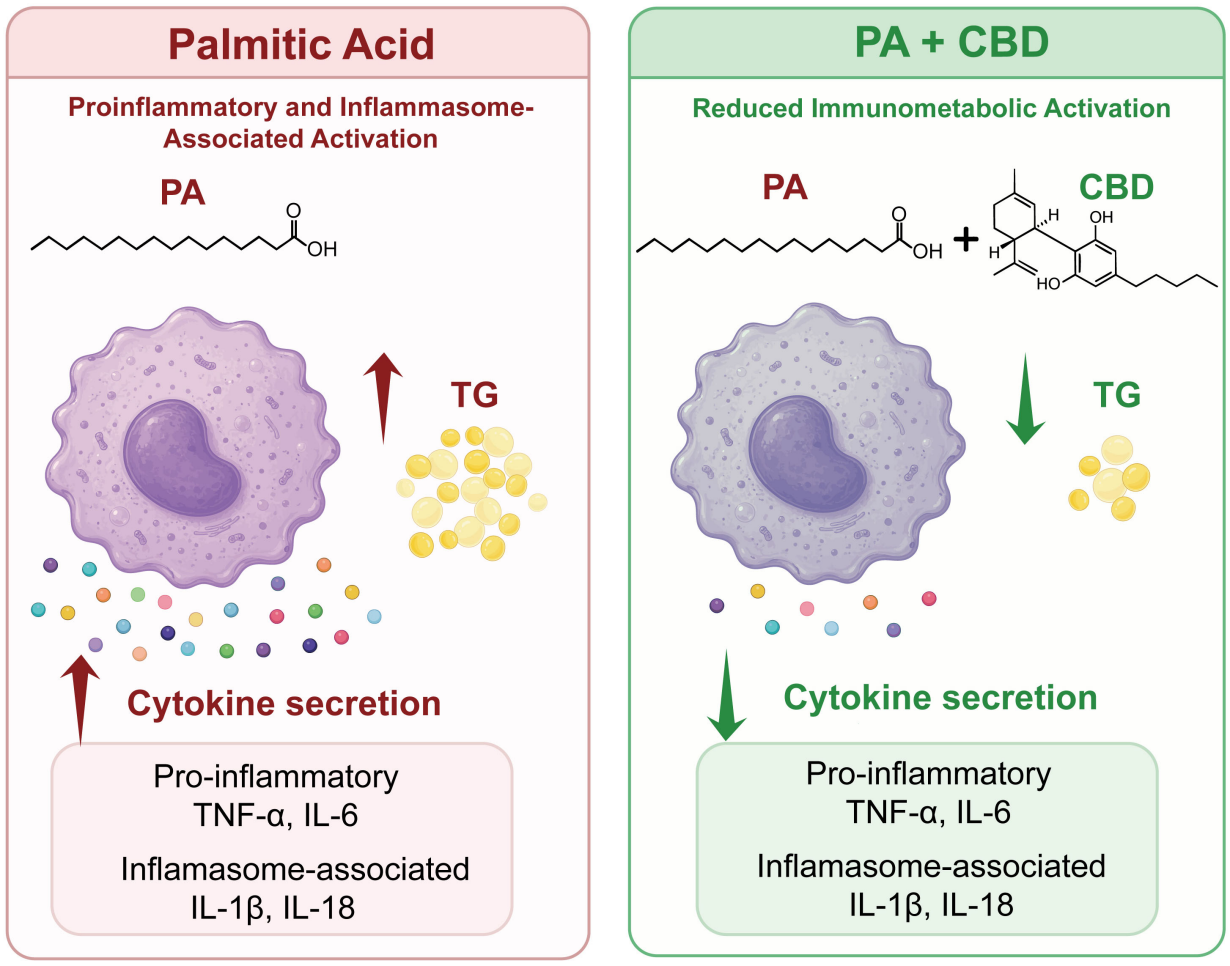
Palmitic acid (PA) induces a pro-inflammatory, lipotoxic phenotype characterized by increased intracellular lipid accumulation, elevated triglyceride (TG) content, and enhanced secretion of pro-inflammatory cytokines (TNF-α and IL-6) and inflammasome-associated cytokines (IL-1β and IL-18). Cannabidiol (CBD) co-treatment reduced intracellular lipid accumulation, triglyceride content, and inflammatory cytokines to levels comparable to those observed in control cells, under the experimental conditions evaluated.

Notably, CBD reduced PA-induced pro-inflammatory cytokine release and partially mitigated intracellular lipid storage without compromising cell viability. These findings indicate that CBD modulates lipid-driven immunometabolic inflammation in a stimulus-dependent manner, with more evident effects under lipotoxic conditions than under restricted endotoxin-induced activation. Overall, this work supports CBD as a context-dependent modulator of immunometabolism response in PMA-differentiated U937-derived macrophages and provides a controlled experimental framework for studying lipid-driven inflammatory dysfunction in U937-derived macrophages (**Figure 4**).

## Data Availability

The original contributions presented in the study are included in the article/**Supplementary Material**. Further inquiries can be directed to the corresponding authors.
