# Supplementary material for: Cannabidiol selectively attenuates lipotoxic immunometabolic inflammation in human macrophages
Source: Front Immunol. 2026 Jul 16;17:1873494. doi: 10.3389/fimmu.2026.1873494 (PMC13421177; doi:10.3389/fimmu.2026.1873494)
Supplement: Supplementary file 1 [file Table1.docx]

Supplementary Material

**Supplementary Table 1.** Cytokine concentrations and statistical comparisons across experimental conditions. Cytokine levels are reported as mean ± SEM for control, LPS, and PA conditions. P values correspond to Dunnett’s post hoc comparisons following one-way ANOVA, in bold those which P < 0.05.

| **P values** | | |  |  |  |  |
| --- | --- | --- | --- | --- | --- | --- |
| **Cytokine** | **Control** | **LPS** | **PA** | **Ctrl vs PA** | **Ctrl vs LPS** | **LPS vs PA** |
| IL-1β | 144818.26 ± 83329.50 | 562764.67 ± 69218.14 | 899854.15 ±317505.97 | **0.0019** | **0.0002** | 0.4587 |
| TNF-α | 268063.56 ±101810.57 | 959460.36 ±140316.57 | 636632.21 ±214710.62 | 0.0991 | **0.0016** | 0.356 |
| IL-6 | 412457.99 ±283147.06 | 1253212.53 ±152342.16 | 1608020.84 ±493133.54 | **0.0029** | **0.0073** | 0.7462 |
| MCP1 | 1259659.96 ±251121.67 | 1366915.77 ±120423.98 | 1880983.20 ±253985.53 | 0.0844 | 0.9223 | 0.2559 |
| IL-8 | 273042.55 ±120248.57 | 829289.03 ±109694.97 | 394758.11 ± 98809.40 | 0.8942 | **0.0067** | **0.0399** |
| IL-10 | 364737.73 ± 52237.81 | 200110.42 ± 28508.37 | 288743.61 ± 51526.04 | 0.5220 | **0.0006** | 0.3818 |
| IL-12p70 | 14373.43 ±896.64 | 18285.22 ±1847.50 | 27432.52 ±6357.31 | 0.0885 | **0.0485** | 0.2535 |
| IL-17A | 5998.22 ±166.16 | 6776.61 ±719.15 | 6420.63 ±163.49 | 0.9999 | 0.4166 | 0.8349 |
| IL-18 | 18520.55 ±1691.07 | 20504.91 ±1218.65 | 37857.14 ±9515.39 | **0.0241** | 0.4885 | 0.115 |
| IL-23 | 16848.10 ±1397.71 | 32697.93 ±8008.87 | 41194.73 ±10914.68 | **0.0125** | **0.0397** | 0.7308 |
| IL-33 | 13380.48 ±2691.90 | 42761.45 ±16481.12 | 42828.69 ±20624.51 | 0.1012 | 0.0642 | 1 |
| IFN-α2 | 24573.54 ±1288.69 | 29455.14 ±2498.49 | 28286.36 ±2167.84 | 0.3753 | 0.0913 | 0.9149 |
| IFN-γ | 18251.89 ±4182.61 | 8820.21 ±270.15 | 8801.70 ±673.91 | 0.3668 | 0.4835 | 1 |
